# Strain Transfer in Surface-Bonded Optical Fiber Sensors

**DOI:** 10.3390/s20113100

**Published:** 2020-05-30

**Authors:** Francesco Falcetelli, Leonardo Rossi, Raffaella Di Sante, Gabriele Bolognini

**Affiliations:** 1 Department of Industrial Engineering-DIN, University of Bologna, 47121 Forli, Italy; francesco.falcetelli@unibo.it (F.F.); raffaella.disante@unibo.it (R.D.S.); 2 IMM Institute, Consiglio Nazionale delle Ricerche, 40129 Bologna, Italy; rossi@bo.imm.cnr.it

**Keywords:** fiber optic sensors, strain transfer, distributed sensing, optical fiber cables

## Abstract

Fiber optic sensors represent one of the most promising technologies for the monitoring of various engineering structures. A major challenge in the field is to analyze and predict the strain transfer to the fiber core reliably. Many authors developed analytical models of a coated optical fiber, assuming null strain at the ends of the bonding length. However, this configuration only partially reflects real experimental setups in which the cable structure can be more complex and the strains do not drastically reduce to zero. In this study, a novel strain transfer model for surface-bonded sensing cables with multilayered structure was developed. The analytical model was validated both experimentally and numerically, considering two surface-mounted cable prototypes with three different bonding lengths and five load cases. The results demonstrated the capability of the model to predict the strain profile and, differently from the available strain transfer models, that the strain values at the extremities of the bonded fiber length are not null.

## 1. Introduction

In recent years, the use of Optical Fiber Sensors (OFS) has spread throughout the scientific community and the industry for their beneficial sensing capabilities in several applications. Among the various advantages of OFS over traditional sensing techniques, their large bandwidth (which allows the transmission of a large amount of information in the same physical line), small size and light weight, immunity to electromagnetic interference (due to their dielectric nature) and durability [1,2] are worth mentioning.

Optical fibers must survive harsh environmental conditions for several in-situ monitoring applications [3,4,5]. In these cases, the addition of multiple coatings is useful to prevent possible damage or breakage of the optical fiber. On the other hand, protective layers usually cause a discrepancy between the strain profile of the structure and the fiber core, introducing uncertainty in the measuring system. The analysis of the strain transfer mechanism, from the structure to the external fiber coatings and the fiber core, is therefore relevant to obtain accurate values of the actual strain in the structure. For this reason, it has attracted the attention of many researchers.

The initial studies on the strain transfer mechanism focused on optical fiber sensors embedded in composite or concrete structures. In [6], Cox introduced the shear lag theory, establishing the fundamentals for the development of future strain transfer models. Subsequently, Claus et al., discussed the behavior of embedded optical fibers during the life cycle of structural components, highlighting the role of the fiber coating in the strain transfer process [7]. Nanni et al., investigated the use of optical fibers for in situ monitoring of concrete structures and demonstrated that the embedding direction (with respect to the applied load) influences the performance of the sensor [8]. Pak studied the strain transfer efficiency of an optical fiber embedded in a host matrix, demonstrating that the strain transfer is maximized when the shear modulus of the coating equals the geometric mean value of the shear moduli of the matrix and fiber [9]. Ansari and Libo developed a complete strain transfer model for an embedded optical fiber with three layers: the fiber core, coating and host structure [10]. They introduced for the first time the shear lag parameter, which condenses the mechanical and geometrical properties of the system. Their model became a reference in the field but was also discussed by other authors for the use of inadequate boundary conditions (BCs), i.e., the complete strain transfer at the fiber midpoint. Li et al., derived an analytical model to predict the strain transfer related to Fiber Bragg Grating (FBG) sensors [11]. The authors demonstrated that the strain at the fiber midpoint does not necessarily match the strain value present in the host material. The BCs were applied at the two ends of the fiber assuming that the normal strain in the fiber core is null. In a subsequent study [12], the same authors proposed a refined strain transfer model considering also the mutual interaction between the fiber and the host material. The shear lag constant was redefined without changing the governing equations and the BCs. The study carried out by LeBlanc et al., analyzed the effect of strain gradients on the reflected spectra in FBG sensors [13]. The strain gradients arise due to the strain transfer phenomenon, causing the peak broadening of the reflected spectrum and affecting the spatial resolution. When FBG sensors are embedded in composite laminates or bonded at high temperatures, transverse stress fields are likely to be present [14]. This condition can lead to peak splitting effects in the reflected spectra, because of the strain-induced birefringence in the optical fiber. These effects should be taken into account in the strain transfer analysis to avoid misinterpretation of the results. Recently, Wang and Xiang studied the behavior of optical fibers embedded in asphalt pavements, making use of the Goodman’s hypothesis to model the interfacial shear stresses [15].

Despite the increasing interest in embedded optical fiber sensors configurations, surface-bonded optical fiber sensors still represent a viable solution for many applications. In this case, the strain transfer model from the structure to the fiber core is asymmetric and the complexity of the analysis increases [16]. Wan et al., made a first parametric analysis of the strain transfer for surface-bonded optical fibers studying the influence of the side width, bonding length, bottom and top thickness of the adhesive [17]. They used the analytical model for embedded optical fiber developed by Li et al. [11], and analyzed its range of validity for the surface-bonded configuration. The complexity of the geometrical layout forced the authors to rely on Finite Element Method (FEM) simulations to determine the shear lag characteristics. Subsequently, Li et al., studied the strain transmission of a surface-bonded FBG sensor [18]. In their work, they derived the strain distribution not only for the FBG sensor but also for the substrate structure, emphasizing their mutual interaction. Her and Huang modeled a segment of a surface-bonded optical fiber with a more complex structure [19]. Their model consisted of four different layers, i.e., fiber core, coating, adhesive and substrate structure, including the possibility of a gap in the adhesive. Feng et al., investigated the strain transfer phenomenon for crack detection purposes using Distributed Optical Fiber Sensors (DOFS) [20]. Billon et al., developed a qualification methodology for DOFS [21]. Unlike previous analytical models, they used a hybrid approach based on the derivation of a mechanical transfer function which is not known a priori and must be computed with the aid of FEM simulations. They also highlighted the importance of considering the interrogator resolution in the strain transfer analysis for crack detection.

Despite the number of available studies, the research is still limited to rather simple configurations. The cited models for surface-bonded optical sensors consider a maximum of four layers. Moreover, in all the considered analytical models the strain at the ends of the bonding length is considered null. This configuration is not truly representative of real experimental setups. In fact, the deformation in the fiber structure does not decrease dramatically to zero. This discrepancy has implications for the BCs applied at the fiber ends and, therefore, may alter the prediction of the strain transfer profile.

In this study a novel strain transfer model is investigated. Seven layers were considered in the analysis, i.e., structure, adhesive, cable jacket, tight tubing, outer and inner coatings and fiber core, in order to provide a through representation of the largest number of possible fiber cable designs. The sensing cable was extended beyond the ends of the bonding length to reproduce the experimental setup more accurately. The model was validated both numerically and experimentally using DOFS, using two cable prototypes developed within the European Horizon 2020 project “Pervasive Ubiquitous Lightwave Sensor—PULSe” [22]. The general aim of PULSe is to develop a cost-effective Brillouin distribute sensing solution based on a synergy of innovative interrogator equipment (exploiting coding techniques [23] and ring lasers schemes [24]), strain sensing cable, data processing software, and open-access market take-up support tools.

In principle, the novel methodology can be applied to any type of optical fiber sensor. Nevertheless, the use of distributed sensors for the validation phase presents some advantages. Distributed sensing offers the possibility to validate the predicted strain transfer along the whole fiber with high resolution. This is particularly critical when dealing with high strain gradients, as is the case of bonded segments. For this reason, it was decided to validate the analytical model with DOFS cables.

Finally, the results are presented and discussed to assess the performance of the novel analytical model for different bonding lengths, load levels, and cable geometries.

## 2. Materials and Methods

**Figure 1:**
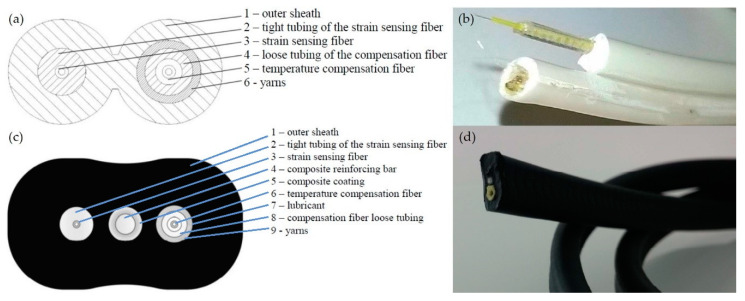
First cable prototype conceptual layout (**a**) and manufacturing design (**b**). Second cable prototype conceptual layout (**c**) and manufacturing design (**d**).

The two fiber sensing cables considered in this study are reported in Figure 1. Both strain sensors are characterized by a multilayered structure. They are equipped with an additional sensing fiber that allows for the compensation of temperature effects. The outer sheath protects the fiber against environmental agents. The intermediate tight tubing ensures further protection and optimizes the mechanical coupling between the fiber and the outer layers. The mechanical decoupling of the temperature sensing fiber from the jacket is obtained by the insertion of aramid Kevlar^®^ yarns and a lubricant. The high modulus Kevlar^®^ yarns attenuate the deformations in the vicinities of the temperature compensation fiber, whereas the silicon lubricant significantly reduces the friction coefficient between the fiber and the outer layer.

The composite reinforcing bar, which is only present in the second cable prototype, is added to avoid severe bending and thus possible breakage of the fiber. On the other hand, due to its coating and the silicon lubricant, it is mechanically decoupled from the outer sheath. This design feature prevents the reinforcing bar to bear a significant amount of axial load instead of the fiber core, which would result in a delay of the strain transfer mechanism. In both cables, the sensing fiber (Corning^®^ SMF-28e+^®^ LL) has a dual-layer coating system made of a primary (or inner) coating and secondary (or outer) coating. The material properties and the geometrical dimensions of the two cables are summarized in Table 1 and Table 2.

The strain transfer phenomenon was studied by bonding the two optical cables on the surface of an aluminum specimen with the material and geometrical properties reported in Table 3.

The methodology adopted in this study consists of:

- Development of the analytical model of the two sensing cables.
- Development of the experimental setup and testing.
- Numerical modeling of the experimental setup.

### 2.1. Analytical Model

The basic analytical model for the two sensing cables is in line with the traditional strain transfer models developed for bare surface-bonded optical fibers. However, in the present case some additional assumptions are considered and different boundary conditions (BCs) are applied. The model is developed under the following assumptions:

A1. All the materials involved in the analysis behave as linear elastic materials and there is perfect bonding at all the layer interfaces.
A2. It is assumed that the fiber core and the cladding behave as a unique homogeneus material which is referred to as “optical fiber”.
A3. The optical fiber coatings, the corresponding tight tubing, the cable jacket, and the adhesive carry only shear stresses. Indeed, the Young moduli of these cable components are at least one or two orders of magnitude smaller than those of the optical fiber and the specimen.
A4. The strain transfer from the structure towards the fiber core depends only on the cable components surrounding the fiber under test. Therefore, referring to Figure 1a,c only the left half of the two cables, where the strain sensing fiber is embedded, was considered in the development of the model.
A5. In the second cable prototype the effect of the reinforced bar is neglected, since, as already said, it is mechanically decoupled from the surrounding cable jacket.

**Figure 2:**
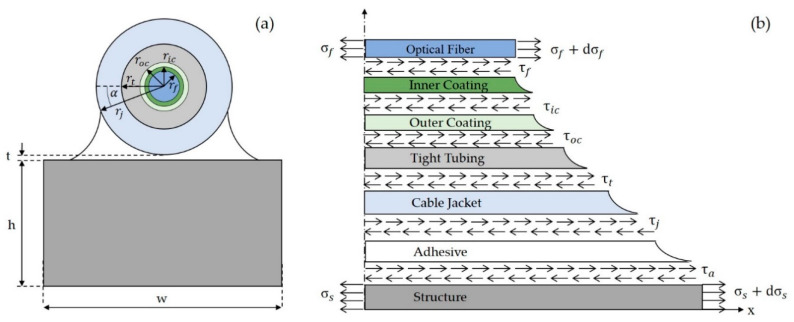
Multilayered model for the two cable prototypes. Cross section (**a**), free body diagram of an infinitely small cable segment (**b**).

Based on the assumption A4 only one half of the cable is considered, modelling its geometry as outlined in Figure 2.

The analysis was carried out using cylindrical coordinates. The axial direction, along the axis of the optical fiber, is denoted with *x*, the radial direction with *r*, whereas ϑ represents the azimuth.

The analysis starts considering an infinitesimal fiber segment and imposing the equilibrium condition:

(1) $$\left(\sigma_{f}+d\sigma_{f}\right)\pi r_{f}^{2}-\sigma_{f}\pi r_{f}^{2}+\int_{0}^{2\pi }\tau \left(x,r_{f}\right)r_{f}d\vartheta \cdot dx=0$$

where, referring to Figure 2, $r_{f}$ represents the optical fiber radius, $\sigma_{f}$ denotes the normal stress in the optical fiber and $\tau \left(x,r_{f}\right)$ is the shear stress at the interface between the optical fiber and the inner coating.

Then, it is possible to extract the shear stress at the optical fiber boundary as follows:

(2) $$\tau \left(x,r_{f}\right)=-\frac{r_{f}}{2}\frac{d\sigma_{f}}{dx}$$

Recalling the assumption A3, the equilibrium condition in the *x* direction of the first layer surrounding the optical fiber, which is the inner fiber coating, leads to Equation (3):

(3) $$\int_{\alpha }^{\pi -\alpha }\tau \left(x,r\right)rd\vartheta \cdot dx-\int_{0}^{2\pi }\tau \left(x,r_{f}\right)rd\vartheta \cdot dx=0$$

The first integral of Equation (3) is defined within the interval [α,π−α], where α represents the angle between the horizontal direction and the line connecting the center of the optical fiber with the top point of the adhesive layer on the cable surface (see Figure 2). In the case of an embedded optical fiber the integration interval would be [0, 2π] as is for the second term of Equation (3). However, for surface bonded optical cables the strain field is not axially symmetric. Hence, the shear stresses in the coating can be expressed as:

(4) $$\tau \left(x,r\right)=\frac{2\pi }{\pi -2\alpha }\frac{r_{f}}{r}\tau \left(x,r_{f}\right)$$

Substituting Equation (2) into Equation (4) one obtains:

(5) $$\tau \left(x,r\right)=-\frac{\pi }{\pi -2\alpha }\frac{r_{f}^{2}}{r}\frac{d\sigma_{f}}{dx}$$

Assumption A1 allows to use Hooke’s law, relating stresses to strains with the constitutive equations:

(6) $$\{\begin{array}{c} \sigma =E\varepsilon \\ \tau =G\gamma \end{array}$$

where E, G, ε and γ represent, respectively, the Young’s modulus the shear modulus, the normal strain and the shear strain of a generic layer of the sensing cable. Based on these parameters, Equation (5) can be rewritten as:

(7) $$\gamma \left(x,r\right)=-\frac{1}{G_{ic}}\frac{\pi }{\pi -2\alpha }\frac{r_{f}^{2}}{r}E_{f}\frac{d\varepsilon_{f}}{dx}$$

where $G_{ic}$, $E_{f}$ and $\varepsilon_{f}$ represent the shear modulus of the inner coating, the Young’s modulus and the normal strain of the optical fiber, respectively.

The shear strain can be expressed under the assumption of small displacements:

(8) $$\gamma \left(x,r\right)=\left(\frac{\partial u}{\partial r}+\frac{\partial w}{\partial x}\right)$$

The radial displacements, *w*, are negligible compared to the axial displacements *u*. Indeed, the radial displacements are mainly induced by the Poisson contraction occurring in the coating and the displacements along the *x* axis are at least one order of magnitude higher than *w*. Hence, substituting Equation (8) into Equation (7) leads to:

(9) $$\gamma \left(x,r\right)≅\frac{\partial u}{\partial r}=-\frac{1}{G_{ic}}\frac{\pi }{\pi -2\alpha }\frac{r_{f}^{2}}{r}E_{f}\frac{d\varepsilon_{f}}{dx}$$

Then, integrating Equation (9) from the outer optical fiber radius, $r_{f}$, to the inner coating boundary, $r_{ic}$ one gets:

(10) $$\int_{r_{f}}^{r_{ic}}\frac{\partial u}{\partial r}dr=\int_{r_{f}}^{r_{ic}}-\frac{1}{G_{ic}}\frac{\pi }{\pi -2\alpha }\frac{r_{f}^{2}}{r}E_{f}\frac{d\varepsilon_{f}}{dx}dr$$

The result of the integration is given by Equation (11), with $u_{ic}$ and $u_{f}$ being the axial displacements of the inner coating and the optical fiber, respectively:

(11) $$u_{ic}-u_{f}=-\frac{1}{G_{ic}}\frac{\pi }{\pi -2\alpha }r_{f}^{2}E_{f}\frac{d\varepsilon_{f}}{dx}\ln\frac{r_{ic}}{r_{f}}$$

Performing the same operation for all the other layers leads to:

(12) $$u_{s}-u_{f}=-\frac{\pi }{\pi -2\alpha }r_{f}^{2}E_{f}\frac{d\varepsilon_{f}}{dx}\left[\frac{1}{G_{a}}\ln\frac{t_{a}}{r_{j}}+\frac{1}{G_{j}}\ln\frac{r_{j}}{r_{t}}+\frac{1}{G_{t}}\ln\frac{r_{t}}{r_{oc}}+\frac{1}{G_{oc}}\ln\frac{r_{oc}}{r_{ic}}+\frac{1}{G_{ic}}\ln\frac{r_{ic}}{r_{f}}\right]$$

where the axial displacement of the structure is denoted with $u_{s}$, whereas $G_{a}$, $G_{j}$, $G_{t}$ and $G_{oc}$ and $r_{j},\;r_{t}$ and $r_{oc}$ are the shear moduli (*G*) and the radii (*r*) of the adhesive, cable jacket, tight tubing and outer coating, respectively. The thickness of the adhesive, $t_{a}$, deserves additional considerations because it is a function of the azimuthal angle ϑ (see Figure 2). In Equation (12), $t_{a}$ is assumed equal to the average adhesive thickness and is calculated as outlined in the following expression:

(13) $$t_{a}=\frac{1}{\pi -2\alpha }\int_{\alpha }^{\pi -\alpha }\left[r_{j}\left(1-\sin\alpha \right)+t\right]d\vartheta =r_{j}+t-\frac{2r_{j}\cos\alpha }{\pi -2\alpha }$$

where *t* is the minimum adhesive thickness (see Figure 2). Substituting Equation (13) into Equation (12), and introducing the shear lag parameter *k*, one gets:

(14) $$u_{s}-u_{f}=-\frac{1}{k^{2}}\frac{d\varepsilon_{f}}{dx}$$

where *k* is defined by the following equation:

(15) $$k=\sqrt{\frac{\pi -2\alpha }{\pi r_{f}^{2}E_{f}\left[\frac{1}{G_{a}}\ln\frac{t_{a}}{r_{j}}+\frac{1}{G_{j}}\ln\frac{r_{j}}{r_{t}}+\frac{1}{G_{t}}\ln\frac{r_{t}}{r_{c}}+\frac{1}{G_{oc}}\ln\frac{r_{oc}}{r_{ic}}+\frac{1}{G_{ic}}\ln\frac{r_{ic}}{r_{f}}\right]}}$$

**Figure 3:**
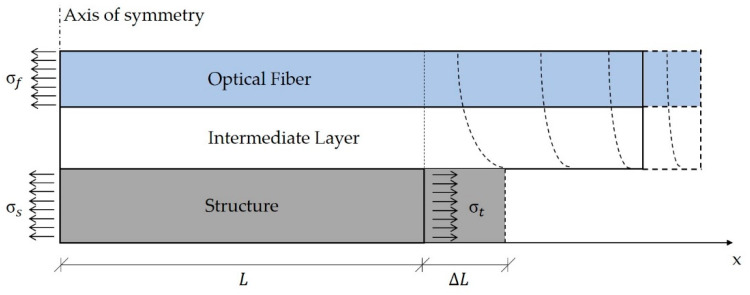
Optical cable response to a ∆L deformation of the structure.

Since the axial strain is defined as the derivative of the longitudinal displacement with respect to the *x* variable, the differentiation of Equation (14) with respect to *x* leads to:

(16) $$\frac{d^{2}\varepsilon_{f}}{dx^{2}}-k^{2}\varepsilon_{f}=-k^{2}\varepsilon_{s}$$

with $\varepsilon_{s}$ being the axial strain of the structure. Equation (16) is a second order linear non-homogeneous differential equation with constant coefficients. Adding up the homogeneous and the particular solutions, one obtains:

(17) $$\varepsilon_{f}\left(x\right)=C_{1}e^{-kx}+C_{2}e^{kx}+\varepsilon_{s}$$

where *C*_1_ and *C*_2_ represent the integration constants whose value can be computed imposing the corresponding BCs. Normally, the strain values at the optical fiber extremities are assumed equal to zero. However, this is not the case in real applications, where the strain does not suddenly reduce to zero, although the cable is not subjected to external loads. Figure 3 represents the actual situation.

Assuming a null strain level in the optical fiber at the two extremities of the bonding length generates a discontinuity in the first derivative of the strain profile which is unlikely to occur. In addition, since the fiber core stiffness is higher than that of the other cable components, the related deformation at the fiber boundaries is expected to be significantly lower with respect to the outer layers. Consequently, the fiber core prevents the cable jacket from stretching whereas the cable jacket tends to stretch the fiber core. This results in a self-equilibrating configuration where the fiber core experiences a tensile load whereas the other cable components undergo a compressive load. Such effect vanishes after few cable diameters (Figure 3) based on the De Saint Venant principle (stresses are free to redistribute along the structure).

In addition, in a surface-bonded cable the two ends tend to bend upwards as a result of the shear strains acting in those sections. If the optical fiber core is not perfectly centered in the cable structure, the misalignment with the neutral axis produces an additional axial load.

Based on these considerations, the BCs applied to Equation (17) are not null and assumed equal to:

(18) $$\varepsilon_{f}\left(\pm L\right)=p\varepsilon_{s}$$

where *L* is half of the bonded length and the *p* parameter symbolizes the percentage of residual strain in the optical fiber core, thus p∈[0,1].

Imposing the BCs defined in Equation (18), the integration constant *C*_1_ and *C*_2_ can be found to be:

(19) $$C_{1}=C_{2}=\frac{\left(p-1\right)}{2}\varepsilon_{s}\sec h\left(kL\right)$$

Then, the substitution of *C*_1_ and *C*_2_ into Equation (17) leads to an expression for the strain profile of the fiber core as a function of *x*:

(20) $$\varepsilon_{f}\left(x\right)=\varepsilon_{s}\left[1+\left(p-1\right)\frac{\cosh\left(kx\right)}{\cosh\left(kL\right)}\right]$$

Equation (20) holds when x∈[−L,L].

For x>L and x<−L, it is assumed that, in accordance with the De Saint Venant principle, the axial strain shows an exponential decay as follows:

(21) $$\varepsilon_{f}\left(x\right)=ae^{-b\left|x\right|}=\{\begin{array}{cc} ae^{+bx} & \mathrm{for}\;x<0\\ ae^{-bx} & \mathrm{for}\;x>0 \end{array}$$

Equation (21) represents an even function in line with the fact that the strain profile should be symmetric with respect to the sensing cable midpoint. The a, b, and p parameters can be determined by fitting the experimental data. However, the authors propose the following methodology to assess their value without any prior test. The b parameter represents the exponential strain decay in the optical cable beyond the bonding length (i.e., x>L ∨x<−L). Hence, an estimate of b can be carried out using the same approach used to determine the shear lag parameter k. However, in this case the adhesive layer is not present and the first term of Equation (3) should be integrated from 0 to 2π since the strain propagates with no preferential direction as in the case of a fully embedded optical fiber. These considerations lead to the following expression for *b*:

$$b=\sqrt{\frac{2}{r_{f}^{2}E_{f}\left[\frac{1}{G_{j}}\ln\frac{r_{j}}{r_{t}}+\frac{1}{G_{t}}\ln\frac{r_{t}}{r_{c}}+\frac{1}{G_{oc}}\ln\frac{r_{oc}}{r_{ic}}+\frac{1}{G_{ic}}\ln\frac{r_{ic}}{r_{f}}\right]}}$$

The other two parameters, *a* and *p*, can be evaluated by imposing the continuity of the strain profile and its derivative at the two extremities, where x=±L. The derivative of the strain profile is estimated differentiating Equation (20) along the *x* axis as follows:

(22) $${\varepsilon_{f}}^{\prime }\left(x\right)=\varepsilon_{s}k\left(p-1\right)\frac{\sinh\left(kx\right)}{\cosh\left(kL\right)}$$

Considering for example the interval x∈[L,+∞] it is possible to write the following system of equations:

(23) $$\{\begin{array}{l} \varepsilon_{f}\left(L\right)=ae^{-bL}=p\varepsilon_{s}\\ \varepsilon_{f}^{\prime }\left(L\right)=-abe^{-bL}=\varepsilon_{s}k\left(p-1\right)\tanh\left(kL\right) \end{array}$$

The system solved for *a* and *b* returns:

(24) $$\{\begin{array}{l} a=\frac{e^{bL}\varepsilon_{s}\tanh\left(kL\right)}{b/k+\tanh\left(kL\right)}\\ p=\frac{\tanh\left(kL\right)}{b/k+\tanh\left(kL\right)} \end{array}$$

Once every parameter of the model is determined and the corresponding strain profile is computed, it is convenient to introduce the so-called effective bonding length $L_{\mathrm{eff}}$, which has been defined in the literature by several authors using various expressions [11,25,26]. In this study, $L_{\mathrm{eff}}$ is defined as the minimum half fiber length to be bonded such that at the midpoint (i.e., x=0) of the fiber core the strain level reaches 95% of the strain present in the structure. Assuming $\varepsilon_{f}\left(0\right)=0.95\varepsilon_{s}$, $L_{\mathrm{eff}}$ can be obtained from Equation (20) as follows:

(25) $$L_{\mathrm{eff}}=\frac{1}{k}\cosh^{-1}\left(\frac{1-p}{0.05}\right)$$

Hence, the higher the shear lag parameter, the lower the corresponding effective bonding length. Moreover, since *p* represents the percentage of strain in the fiber core at x=±L with respect to the strain present in the structure, it can be stated that high values of *p* entail lower values of $L_{\mathrm{eff}}$. An alternative conservative approach would be to apply Equation (25) with p=0.

#### 2.1.1. Cable-Specimen Interaction

It is worth to consider in the analysis the mutual interaction between the sensing cable and the structure if the former is particularly stiff with respect to the latter. Referring to Figure 3, it is possible to relate the theoretical strain in the structure with the actual strain, i.e., the result of the reciprocal interaction between the sensing cable and the structure. The equilibrium condition for the system is given by:

(26) $$\sigma_{t}A_{s}=\sigma_{s}A_{s}+\sigma_{f}A_{f}$$

where $\sigma_{t}$ is the true stress applied to the structure, $\sigma_{s}$ is the corresponding actual stress, $\sigma_{f}$ is the stress acting in the optical fiber, whereas $A_{s}$ and $A_{f}$ are the cross section of the structure and the optical fiber, respectively. Exploiting the Hooke’s law and substituting the values of the relative cross sections one has:

(27) $$E_{s}\varepsilon_{t}hw=E_{s}\varepsilon_{s}hw+E_{f}\varepsilon_{f}\pi r_{f}^{2}$$

where *h* and *w* are the two cross section dimensions of the structure (Figure 2), and $E_{s}$ is its modulus of elasticity. Solving for the actual longitudinal strain in the substrate structure, $\varepsilon_{s}$, leads to:

(28) $$\varepsilon_{s}=\varepsilon_{t}-\varepsilon_{f}\frac{E_{f}\pi r_{f}^{2}}{E_{s}hw}$$

Hence, when the cable stiffness is not negligible with respect the host structure, the mutual interaction must be considered.

#### 2.1.2. Interrogator Resolution

The interrogator resolution has an impact on the measured strain profile, $\varepsilon_{m}$. In [27] J.M. Henault et al., estimated the interrogator effect on the strain transfer mechanism by convolving the strain profile in the fiber core, $\varepsilon_{f}$, with a rectangular function $\Pi_{i}\left(x\right)$. The interval width of $\Pi_{i}\left(x\right)$ corresponds to the resolution of the measuring system. Hence, the filtering operation due to the interrogator can be expressed by:

(29) $$\varepsilon_{m}\left(x\right)=\varepsilon_{f}\left(x\right)⊗\Pi_{i}\left(x\right)$$

where the symbol ⊗ denotes the convolution operator. For a consistent comparison between the analytical and the experimental data it is necessary to filter the analytical model results according to Equation (29).

### 2.2. Experimental Methodology

#### 2.2.1. Sensing Principle

The experimental activity was carried out at the Materials Structures Technologies Research Laboratory (MaSTeR Lab, University of Bologna, Bologna, Italy). A distributed fiber sensing technique was used in order to obtain pointwise data on the deformation occurring in the fiber core. In particular, the measurements were performed using a LUNA Optical Backscatter Reflectometer™ (OBR 4413). The working principle is based on the Swept-Wavelength Interferometry (SWI) technique, and the interested reader can find additional information in several references [28,29,30].

Using the same mathematical description adopted for FBG sensors, a change in temperature, Δ*T*, or mechanical strain, *ε*, entails a shift in the reflected wavelength, $\Delta \lambda_{R}$, or spectrum, $\Delta \nu_{R}$, as follows:

(30) $$\frac{\Delta \lambda_{R}}{\lambda_{R}}=\frac{\Delta \nu_{R}}{\nu_{R}}=K_{T}\Delta T+K_{\varepsilon }\varepsilon$$

where $K_{T}\;\mathrm{and}\;K_{\varepsilon }$ denote the temperature and strain calibration coefficients, respectively.

The strain transfer model developed in Section 2.1 does not consider the effect of temperature. Consequently, the experiments were conducted at constant ambient temperature to make the term $K_{T}\Delta T$ negligible and filter any undesired thermal effect. The accuracy of the measurement depends on the level of accuracy of the strain calibration coefficient which in turns depends on the photo-elastic coefficient, $\rho_{\varepsilon }$:

(31) $$K_{\varepsilon }=1-\rho_{\varepsilon }$$

The photo-elastic coefficient is defined as:

(32) $$\rho_{\varepsilon }=\frac{n_{eff}^{2}\;}{2}\left[p_{12}-\nu \left(p_{11}+p_{12}\right)\right]$$

where ν is Poisson’s ratio of the fiber core and $p_{11}\;\mathrm{and}\;p_{12}$ are the components of the strain optic tensor [31]. All these parameters are affected by uncertainty depending on the concentration of the dopant species in the fiber core and the composition of the outer layers such as the cladding and the coating [32]. For standard silica fibers with germanium doped core it is common to approximate $\rho_{\varepsilon }$ to 0.22 [2,30].

Assuming a constant temperature, it is possible to derive the expression linking the strain and spectral shift:

(33) $$\varepsilon =-\frac{\bar{\lambda }}{cK_{\varepsilon }}\Delta \nu_{R}=\alpha \Delta \nu_{R}$$

where $\bar{\lambda }$ is the scan centre wavelength ($\bar{\lambda }=1306$ nm for the OBR 4413) and α denotes the static sensitivity of the measuring system. Considering the standard value of 0.22 for the photo-elastic coefficient, leading to a value of 0.78 for $K_{\varepsilon }$, and substituting the values of $\bar{\lambda }$ and c into Equation (34), one obtains α=−5.59 με/GHz.

However, in this study the authors decided to perform a preliminary calibration procedure in order to reduce the uncertainty associated with the estimation of α for a more accurate strain transfer analysis.

#### 2.2.2. Calibration

**Figure 4:**
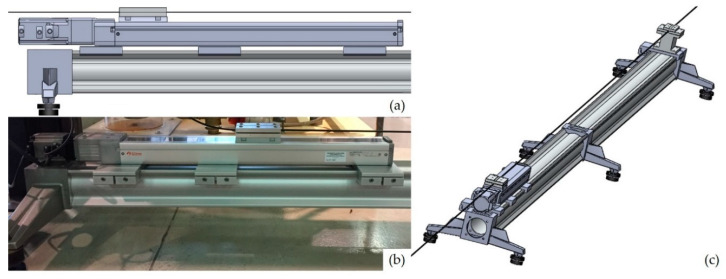
Linear actuator schematic (**a**) and picture (**b**); test rig assembly representation (**c**).

In order to perform the calibration of the two sensing cables, a specific test rig with a high-precision linear actuator was developed (see Figure 4).

The test rig is composed of two rails with a length of 2 m each. They are connected and supported by 3 T-stand elements (max. load of 110 kg) to ensure stiffness, ground levelling and vibration insulation to the structure. The rails have a bending tolerance and a twisting tolerance of 0.8 mm/m and 0.75°/m, respectively.

The linear actuator, with a travel range of 500 mm and a resolution of 0.02 mm, is mounted on one extremity of the test rig allowing to test an optical fiber cable with a length of 3.5 m. The displacement of the moving table surface is regulated using a closed loop controller that is connected to a laptop by means of an USB interface.

**Figure 5:**
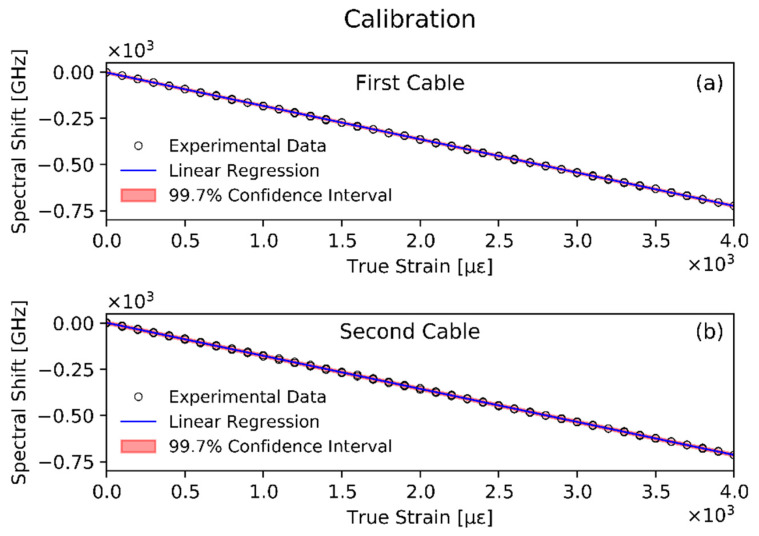
Calibration data for the first (**a**), and the second (**b**), cable prototypes.

The number of points, *n*, considered for the calibration was 80:40 in the forward path (increasing load) and 40 in the backward path (decreasing load). A maximum strain of 4000 με was achieved with steps of 100 με corresponding to a ΔL movement of the translation stage of 0.35 mm. This ΔL value is well above the resolution limit of the linear actuator (0.02 mm), which is key to obtain accurate data. As shown in Figure 5, the calibration results demonstrated a good linear relationship between the spectral shift and the strain, with a negligible hysteresis.

The experimental data were interpolated with a linear regression analysis and the slopes of the two lines were $-0.181\pm 5.8\cdot 10^{-4}\;\mathrm{GHz}/\mu \varepsilon$ and $-0.179\;\pm 7.4\cdot 10^{-4}\mathrm{GHz}/\mu \varepsilon$, respectively, with a confidence interval of three standard deviations.

The corresponding static sensitivity coefficients for the first and second cable prototypes were found to be $\alpha_{1}=-5.52\pm 1.8\cdot 10^{-2}\;\mu \varepsilon /\mathrm{GHz}$ and $\alpha_{2}=-5.59\pm 2.3\cdot 10^{-2}\mu \varepsilon /\mathrm{GHz}$, respectively.

Behind this calibration methodology there is the implicit assumption that the relation linking the strain and the spectral shift is linear. In principle non-linear effects can be present but, in this study, they were not considered. The effect of the quadratic term on the determination of the static sensitivity coefficient, also known as the strain gage factor, is analyzed in [33]. If non-linearities are taken into account, the results show a deviation from the original definition of 0.55%. The quadratic term can therefore be reasonably neglected.

The final step in the calibration procedure is to select a proper spatial shift resolution, ∆x. In principle, the minimum spatial resolution achievable with the interrogator equipment can be computed as:

(34) $$\Delta x_{min\;}=\frac{\lambda_{1}\lambda_{2}}{n_{\mathrm{eff}}\Delta \lambda }$$

which, considering a wavelength scan ranging from 1299.03 nm to 1313.96 nm and an effective refractive index $n_{eff}=1.4676$, leads to a value of 0.078 mm. However, this spatial resolution value is not feasible in practice for stable and accurate measurements. The choice of ∆x depends on two main considerations. First, the number of data points used to compute the Fourier transform and then the cross-correlation is proportional to ∆x. If the number of points involved in the spectral shift computation is insufficient, the noise level increases. Second, the presence of significant variations of the local spectral shift (i.e., high strain gradients) can cause measurements instabilities because of the correlation peak broadening [29]. The best value of ∆x is therefore the result of a compromise between these two considerations. The method used in this study consisted in performing several measurements at constant strain, fixing the translation stage position, and gradually increasing the spectral shift spatial resolution. For each measurement, the standard deviation of the relative spectral shift was computed. A value of 20 mm for ∆x was found to be the best compromise.

#### 2.2.3. Experimental Setup

**Figure 6:**
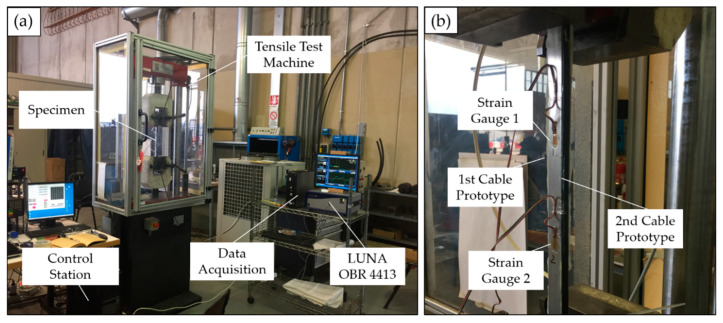
Experimental setup (**a**), and clamped specimen (**b**).

The two cable prototypes were bonded using an epoxy adhesive (LOCTITE^®^ EA 9466™) on the surface of an aluminum specimen. The material and geometrical properties of the adhesive used for the two cables were reported in Table 1 and Table 2, whereas Table 3 summarizes the main characteristics of the specimen. Three different values of the bonding length *L* were considered in the experiments, i.e., 210, 240, and 270 mm, for five applied loads *F* of 5, 10, 15, 20, and 25 kN. Besides the evaluation of the influence of these parameters on the strain transfer, this parametric analysis aims at assessing the presence of non-linear effects. In other words, the study also investigates whether the shear lag parameter depends on the applied load F, hence on the strain value in the structure. Figure 6a illustrates the experimental setup used for the experiments. Two electrical strain gauges, visible in Figure 6b, were fixed on the specimen and used as a reference to estimate the longitudinal strain value. The two optical cables were bonded along the lateral side of the specimen to ensure smooth radii of curvature in the proximity of the specimen clamping areas. In fact, if the optical cables had been attached on the front sides of the specimen, a small curvature radius would have been required at the end of the bonded segment, before the clamps of the tensile test machine.

### 2.3. Numerical Model

**Figure 7:**
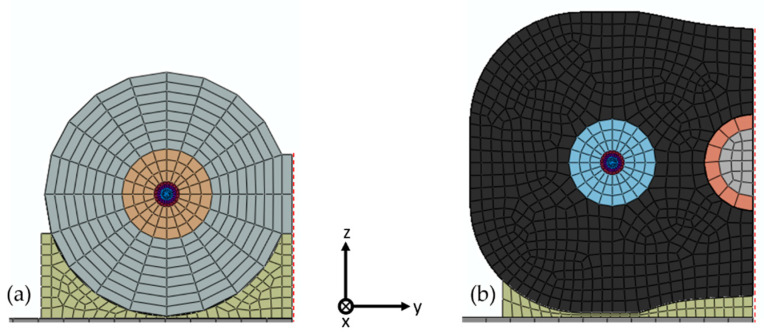
Meshed numerical models with a material-dependent mapping color. First cable prototype (**a**) and second cable prototype (**b**).

The numerical model was developed to validate the proposed analytical model with additional data and provide verified modelling strategies for a complex fiber cable. The Abaqus/CAE^TM^ was used to this aim. In particular, since strain transfer analysis can be considered as a static problem, Abaqus/Standard was chosen as solver. All the model parts were meshed using the C3D8R element type. A preliminary analysis using a microscope was carried out to analyze the cross section of the two sensing cables and thus estimate their effective shape. The material properties and dimensions of the two cables and the specimen were defined according to Table 1, Table 2 and Table 3, respectively. Figure 7 shows the cable cross section of the two meshed models and the reference frame (*x* axis pointing inward).

The analysis was performed applying two symmetry BCs to the two cables in order to simulate one quarter of the model, thus minimizing the computational cost. The first symmetry BC was applied to the cable cross section at the midpoint (x=0). This condition is obtained by posing the displacement along the *x* direction equal to zero and fixing the rotation with respect to the other directions. The other symmetry BC was applied on the x-z plane in correspondence of the dashed red lines in Figure 7, assuming a zero displacement along the y direction and no rotation with respect the *x* and *z* axes.

In both numerical models, the adhesive layer was connected to the structure and cable jacket using a tie constrain between the respective surfaces. In the second numerical model, shown in Figure 7b, the reinforcing bar was coupled to the outer layer with a frictionless connection.

Following the testing procedure outlined in Section 2.2.3, six numerical models were generated. Due to the first symmetry BC, for each cable the simulations were carried out for L equal to 135 mm, 120 mm and 105 mm. In order to take into account the strain variation beyond the bonding length, in the numerical model the two cables were extended by 50 mm, which is more than 10 cable diameters in both cases. This choice is the result of a tradeoff between minimizing the computational cost of the simulation and avoiding any alteration of the strain transfer in the extended region, i.e., for x>L.

The different strain levels were imposed applying a fixed displacement along the *x* direction, ∆u, in the specimen cross section at the end of the bonding length (x=L). The value of ∆u for the different load cases was obtained from the average strain values measured by the strain gauges, $SG_{avg}$, and the corresponding bonding lengths.

Finally, in order to compare the results with the experiments, the computed strain profile along the fiber was convolved with the interrogator resolution according to Equation (29). The latter corresponds to the shift resolution, ∆x, selected in the data processing area of the OBR 4413 system.

## 3. Results and Discussion

**Figure 8:**
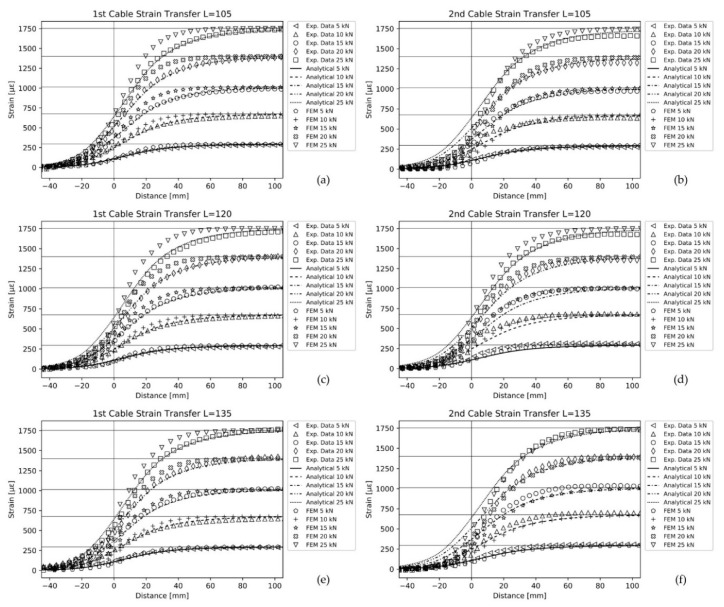
Comparison between experimental data, numerical results and analytical model.

The experimental data were compared with the numerical results and those obtained from the analytical model. The comparison is shown in Figure 8, for the two cable prototypes at the three bonding lengths selected for the analysis. The horizontal lines in each diagram correspond to the average of the strain values measured by the two strain gauges mounted on the central part of the specimen, which is subjected to a constant axial strain. The vertical line indicates the point where the bonded region of the cable starts. Figure 8 shows only one half of the strain profile exploiting the symmetry with respect to the fiber midpoint.

As already illustrated, the analytical results for each subcase were obtained from Equation (28), whereas the parameters *a*, *b* and *p* were estimated following the methodology presented in Section 2.1.

The numerical data were also obtained from Equation (28) but using the deformation values computed along the fiber core in the FEM models.

Referring to Figure 8, it is possible to observe a good qualitative agreement between the novel analytical model and the experimental and numerical data. The analytical model did not require any a priori knowledge on the shear lag parameter, *k*, or the percentage of residual strain in the optical fiber at the end of the bonding length, *p*, and the two constants *a* and *b*, which describe the exponential decay of the longitudinal strain in the regions where the cables were not bonded to the structure. The input data in the model were just the mechanical properties and the geometrical characteristics of the two sensing cables. Nevertheless, if it is possible to perform a preliminary experimental campaign to characterize the cable, the different parameters can be tuned for an optimal matching with real experimental data. Here, the authors discuss the result of the analytical model obtained without any a posteriori tuning, to assess its performance based on the deviation with respect to the numerical and experimental data.

The computed shear lag parameters for the first and the second optical cables were found to be $k_{1}=0.049\;{\mathrm{mm}}^{-1}$ and $k_{2}=0.039\;{\mathrm{mm}}^{-1}$, respectively. Then, the application of Equation (25) led to $L_{\mathrm{eff}1}=66\;\mathrm{mm}$ and $L_{\mathrm{eff}2}=85\;\mathrm{mm}$.

Considering the results obtained for the first cable prototype, it can be seen that there is a good agreement between the analytical model and the experimental data, in both regions, before and after the vertical line, at all the studied load cases.

Regarding the numerical results, they tend to overestimate the strain profile in the bonding region. The numerical model seems to behave as if the cable was stiffer than the one used in the experiments. This discrepancy can be attributed to the uncertainty of the geometrical model used in the simulations, leading to a higher transfer rate. In particular, the adhesive shape plays a key role in the strain transfer mechanism. An inaccurate representation of its shape and thickness may cause a discrepancy between the analytical and experimental data. On the contrary, in the second cable prototype this effect is less evident and occurs only at the highest loads, which is instead a possible sign of nonlinearity in the behavior of the second cable. The modelling of the adhesive layer for the first cable (Figure 7a) is more complex if compared to the second (Figure 7b). In fact, in the first case the adhesive thickness varies significantly along the *y* direction, whereas in the second case is almost constant. The authors believe that this could be a possible explanation for such discrepancy.

The predictions made for the second cable were also satisfactory from a qualitative point of view, but some considerations should be addressed.

In the bonded region, there is a dependency of the results on the different load cases, most likely indicating a nonlinear behavior of the sensing cable. In particular, the analytical model tends to underestimate the strains compared to the experimental data, but not for the highest loads (20 and 25 kN). It seems, therefore, that the strain transfer efficiency of the real cable decreases as the load increases. The fact that the shear lag parameter, *k*, depends on the strain value in the structure, $\varepsilon_{s}$, can be attributed to the first assumption, A1, made for the analytical model. In the second cable prototype the cable jacket is made of a plastic material (i.e., Ethylene-Propylene Diene Monomer) which may start to diverge form the linear behavior at high strain values. Moreover, in real applications there could be micro-slipping between the layer interfaces at the highest loads, leading to a reduction of the effective shear lag parameter.

On the contrary, in the region before the bonded fiber segment, the analytical model predicts higher strain levels. In the authors’ opinion, this effect can be attributed to the fifth assumption, A5, in the analytical model development. The A5 hypothesis was made to simplify the analysis which is already complex in nature. However, if the reinforced bar is not completely disconnected from the system, as it should be, it can bear a portion of axial load due to internal friction between the layers. Consequently, the composite reinforcing rod partially unloads the optical fiber core at the end of the bonded fiber segment.

These considerations can also be used to interpret the comparison between the numerical and experimental data in this region. At the highest loads the cable experiences an increased contraction in the radial direction, which may cause possible grip between the rod and the cable jacket and therefore a likely nonlinearity.

It is worth highlighting that all the experimental results are indeed different from zero at the starting point of the bonding region. This result indicates that the BCs of Equation (18) are well-posed. One could argue that the spreading of the strain profile beyond the bonded area is due to the resolution limit of the interrogator, acting as a filter. Since the convolution operation with a rectangular function extends the strain profile no further than half of the interrogator resolution, the strain should reduce to zero within 10 mm from the vertical line (Figure 8). However, all the strain values measured experimentally and reported in Figure 8 became null well after that value, thus confirming the validity of the approach. In addition, the numerical simulations showed that the results remain practically unchanged even if the resolution is assumed equal to an infinitely narrow window. Considering null strain values at the two bonded fiber ends produces therefore a wrong localization of the bonded region starting point. Referring to Figure 8, the starting point would be placed 40 mm before the actual one if it had to be the point where the strain is null. The proposed analytical model allows a different interpretation of the results, i.e., the starting point of the bonded region should coincide with the inflection point of the strain profile. The inflection point represents the real discontinuity in the system, and it can be useful when dealing, for example, with disbonding or cracks, to locate the exact damage position.

**Figure 9:**
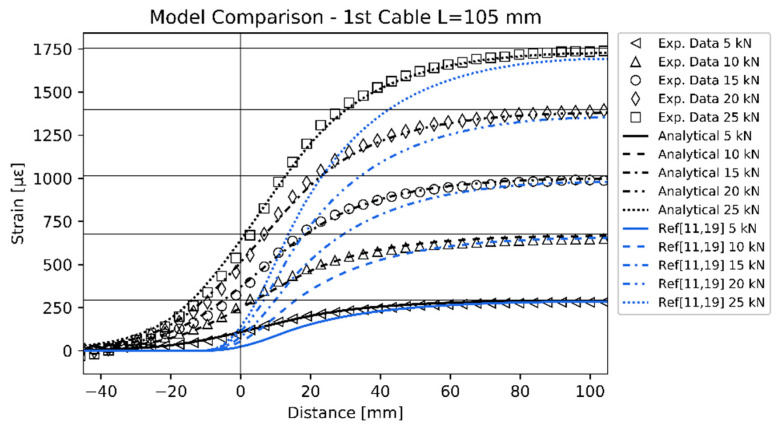
Comparison between the novel analytical model, experimental data and analytical models of references [11,19] using k = $0.049\;{\mathrm{mm}}^{-1}$ and neglecting the reciprocal interaction with the specimen.

The proposed analytical model was then compared with other analytical models related to previous studies (Figure 9).

In particular, even though there are several strain transfer models available in the literature, they are governed by the same differential equations and differ from each other in the way they define the shear lag parameter and the reciprocal interaction with the structure. Thus, neglecting the reciprocal interaction with the structure (i.e., assuming that the structure stiffness is much higher than the cables stiffness), and using the k values obtained in this study for the two optical cables, it is possible to make a consistent comparison with previous studies. Here, the models of Li et al. [11] and Her and Huang [19] are considered. These models, under the previously stated conditions, are consistent with Equation (20) when p=0. The comparison was made considering as a benchmark the experimental results related to the first cable prototype at a bonding length equal to 105 mm. The reason of this choice depends on two considerations. First, for a consistent comparison the first cable prototype is more suitable thanks to its quasi-linear response and relatively simple structure. Second, the shortest length between (105, 120, 135 mm) was chosen because it allows to focus more on the transient region. Indeed, all the models tend to converge for longer bonding lengths and the transient region becomes less visible. The interrogator resolution was taken into account in the previous models applying Equation (29) to their predicted strain profiles.

It is evident from Figure 9 that the previous analytical models did not take into account the transient region, whereas the proposed model is able to predict it. As already stated, from a mathematical point of view the main difference lies in the *p* parameter, which in turn depends on the “*b/k*” ratio (described in Equation (24)). The *b* parameter was defined as the shear lag coefficient of the cable without the effect of the adhesive (because it is related to the free-bonding region). It is possible to infer that the higher the “*b/k*” ratio, the lower is *p,* for similar values of *k* and L. For bare optical fiber *b* increases. The *k* parameter also increases but less, since it takes into account the adhesive effect as well. As a result, for bare optical fiber the “*b/k*” ratio increases and *p* decreases. In this situation the proposed model behaves similarly to those available in the literature. Considering complex optical fiber cables, *b* decreases due to the additional layers. Accordingly, *k* also decreases but again less, due to the adhesive terms in its definition. Consequently, “*b/k*” decreases leading to higher *p* values.

This is the reason why in this study, where complex optical fiber cables were considered, the discrepancies with respect the traditional models were evident. Since previous models were tested with standard optical fibers without a protective cable jacket, their predictions agreed better with experimental data.

## 4. Conclusions

The study focused on the development of a novel analytical model to study the strain transfer phenomenon in multilayered surface-bonded sensing cables. Two cable prototypes developed under the EU-funded PULSe project were investigated under different loading conditions at varying bonding lengths. SWI based on Rayleigh backscattering was adopted to measure the strain profile, allowing the determination of the strain profile shape even in presence of strain gradients at the ends of the sensing fiber segment.

The model considered a more complex cable structure with seven layers and non-null BCs at the end of the attachment area with the aim of reproducing the configuration found in real application cases.

The validation was carried out both numerically and experimentally, demonstrating the capability of the model to predict the strain profile, also in comparison with previous studies.

The authors discussed also the discrepancies arising from possible nonlinear effects, suggesting that further studies would improve the understanding of the strain transfer mechanism for surface-bonded optical fiber sensors in this case.

## Figures and Tables

**Table 1:** Material properties and geometrical dimensions of the first sensing cable.

Sensing Cable: I	Cable Components					
	Optical Fiber	Inner Coating	Outer Coating	Tight Tubing	Cable Jacket	Adhesive
Material	Silica	“Soft” Acrylate	“Stiff” Acrylate	Polyamide	LDPE ^1^	Epoxy
Young’s Modulus [GPa]	21.7	1.30 ∙10^−3^	1.55	2.5	0.2	1.72
Shear Modulus [GPa]	8.89	4.36 ∙10^−4^	0.54	0.9	0.07	0.65
Outer Radius [μm]	62.5	95	125	450	1200	n/a

^1^ Low Density Polyethylene.

**Table 2:** Material properties and geometrical dimensions of the second sensing cable.

Sensing Cable: II	Cable Components					
	Optical Fiber	Inner Coating	Outer Coating	Tight Tubing	Cable Jacket	Adhesive
Material	Silica	“Soft” Acrylate	“Stiff” Acrylate	LDPE ^2^	EPDM ^3^	Epoxy
Young’s Modulus [GPa]	21.7	1.30 ∙10^−3^	1.55	0.2	7.8 ∙10^−3^	1.72
Shear Modulus [GPa]	8.89	4.36 ∙10^−4^	0.54	0.07	2.7 ∙10^−3^	0.65
Outer Radius [μm]	62.5	95	125	450	1800	n/a

^2^ Low Density Polyethylene; ^3^ Ethylene-Propylene Diene Monomer.

**Table 3:** Material properties and geometrical dimensions of the host structure.

Specimen					
Material	Young’s Modulus [GPa]	Shear Modulus [GPa]	Thickness [mm]	Width [mm]	Length [mm]
Aluminum 7075—T6	71.7	26.9	8	20	300
